# Enhancing Resident Engagement and Oral Board Preparation Through a Flipped Classroom Model for the American Board of Surgery

**DOI:** 10.7759/cureus.89992

**Published:** 2025-08-13

**Authors:** Anthony J Duncan, David R Velez, Khaled Zreik, Stefan W Johnson

**Affiliations:** 1 Department of Surgery, University of North Dakota School of Medicine and Health Sciences, Grand Forks, USA; 2 Surgical Critical Care, Sanford Medical Center, Fargo, USA

**Keywords:** examinations, graduate medical education, learning, specialty boards, teaching methods

## Abstract

Background: The American Board of Surgery certification represents the culmination of surgical training, signifying a surgeon's competency and readiness for practice. Residents are less familiar with the oral examination format and often find it more challenging than multiple-choice assessments. Our aim was to evaluate a curriculum change integrating structured oral examination scenarios into our weekly didactic sessions.

Methodology*: *This study evaluated a curricular change performed in 2022. Two approaches were used to assess the impact of this change. A survey was conducted before and one year after the implementation to evaluate subjective perceptions of the new format. We also compared first-time pass rates for the American Board of Surgery Qualifying and Certifying exams before and after the change.

Results: The incorporation of oral scenarios led to increased resident engagement and satisfaction. Residents expressed a preference for the new format and reported feeling better prepared for the American Board of Surgery Certifying Exam. This study also showed a trend toward improved first-time pass rates.

Conclusions: A portion of surgical training is dedicated to preparing residents for the annual examinations and, indirectly, the Qualifying Examination. Given the lower pass rates for the Certifying Examination, surgical training programs should allocate additional resources toward comprehensive board preparation. The addition of oral examination scenarios into routine didactics may serve as an effective strategy to better equip residents for their board certification process.

## Introduction

The traditional teaching model delivers content in a classroom setting, often through podium-style slide lectures, with the expectation that residents will reinforce the material independently. This passive learning approach has proven less effective for millennial and generation Z learners, who tend to struggle with engagement and retention in this format [1]. They tend to learn more effectively through interactive small-group discussions and real-time feedback, which enhance engagement and retention [2]. Modern didactic formats, such as the *flipped classroom* and *team-based learning*, have been introduced to address this issue [3-5]. The flipped classroom presents content at home and applies it in class with immediate feedback, while team-based learning emphasizes individual preparation and peer interaction. Traditional didactic lectures also typically last an hour, despite adult learners having a 15- to 20-minute attention span [6,7]. Lecture effectiveness declines after 20 minutes due to limitations in a person’s working memory and interference, with engagement and motivation playing a critical role in information retention [6].

In the United States, 96% of surgical residency programs use the Surgical Council on Resident Education (SCORE) curriculum, an online portal offering module topics, textbook chapters, and practice questions. SCORE also provides This Week in SCORE (TWIS), a two-year curriculum with weekly topics and quizzes [8]. The curriculum aims to prepare residents for the American Board of Surgery (ABS) Certifying Exam (CE) and Qualifying Exam (QE). The ABS-QE, or *written boards*, has a first-attempt average pass rate of 95% over the past five years, while the ABS-CE, or *oral boards*, has a lower pass rate of 83%-89% [9]. This discrepancy is likely due to the frequency of multiple-choice questions (MCQs) practice during residency. Residents take the ABS In-Training Examination (ABSITE) annually, which consists of MCQs similar to the ABS-QE. Most residents dedicate one to two months per year to studying, using commercially available question banks [10]. By the time the ABS-QE is administered, residents are highly familiar with its format, having answered thousands of similar MCQs throughout their residency training, medical school, and undergraduate studies. In contrast, oral board-style practice is less frequent and can require faculty involvement. While programs provide oral review sessions and occasional mock oral exams, these require substantial faculty time and resources. Consequently, residents graduate with significantly less exposure to oral board-style questioning compared to MCQs.

Modern didactic formats, such as the flipped classroom and team-based learning, have been associated with improved ABSITE performance [3]. Significant effort is dedicated to ABSITE preparation and, indirectly, the ABS-QE, while fewer resources focus on the ABS-CE, despite its lower pass rates. Given these challenges, we implemented a change in our weekly didactic format beginning in the 2021-2022 academic year. This study aims to assess the outcomes and resident satisfaction following our institution’s transition to a flipped classroom model for preparing both the ABS-QEs and ABS-CEs.

## Materials and methods

Study intervention

Our institution incorporates SCORE and TWIS into its curriculum, and in the 2021-2022 academic year, we implemented a modification to our weekly didactic format. Before our intervention, a single resident delivered a 45- to 60-minute slide-based presentation on the weekly TWIS topic using a traditional teaching model. Our intervention revised format divided the hour into two segments. First, junior residents (PGY-1 to PGY-3) delivered a concise 20-25-minute PowerPoint presentation on the weekly TWIS topic. This was followed by a 20-25-minute oral board-style review, where senior residents (PGY-4 and PGY-5) engaged junior residents in structured oral board scenarios. Each week, one junior and one senior resident were assigned to prepare and deliver the content.

Data collection and analysis

The new format was first evaluated subjectively through a resident survey. Before implementing the change, a survey was sent to all residents, assessing satisfaction with the existing TWIS review format using a 5-point Likert scale. Residents were asked: “How satisfied are you with our current TWIS review format?” with response options ranging from (1) Very Dissatisfied to (5) Very Satisfied. They were also asked, “What percentage of TWIS reviews keep your attention and engagement?” with response options of 0% (never pay attention), 25% (occasionally listen but often focus on other tasks), 50% (engaged in about half of the reviews), 75% (usually pay attention but sometimes get distracted), and 100% (fully engaged in every review). After ABSITE, the same survey was distributed to residents the following year to assess the impact of the new format. In addition to the original questions, the post-change survey included: “Which TWIS review format do you prefer?” with options of traditional PowerPoint lectures or the new split format, and “Do you feel these reviews better prepare you for oral board-style questioning?” with a yes or no response (Appendix).

The new format was then evaluated objectively by comparing ABS-QE and ABS-CE first-time pass rates. In order to ensure the privacy of graduates, individual results were compiled by the residency program director and given to the study team in an aggregate fashion. Through a retrospective review, we compared performance from the two years before the format change (Graduation years 2020-2021) to the two years after (Graduation years 2022-2023).

The 5-point Likert scale survey results were compared using a Mann-Whitney U test. ABS-QE and ABS-CE were evaluated percentage of residents who achieved first-time pass and were compared using a Fisher’s Exact test. Statistical significance was set at *P *< 0.05.

The study was evaluated and deemed to be exempt by our institutional review board.

## Results

Response rate for both surveys was 80% (*n *= 20). In the pre-change survey, 2 (10%) residents reported 0% attention and engagement, 8 (40%) were engaged for only 25% of the lectures, 4 (20%) for 50%, 6 (30%) for 75%, and none were fully engaged (100%). Regarding satisfaction with the traditional didactic format, 2 (10%) were very dissatisfied, 4 (20%) dissatisfied, 14 (70%) neutral, 2 (10%) satisfied, and none were very satisfied.

**Figure 1:**
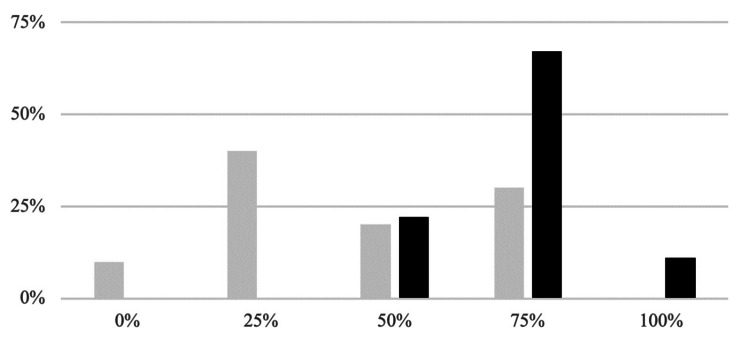
Percentage of time residents were engaged: traditional didactic format vs. split review format. Gray, pre-intervention; black, post-intervention

In the post-change survey, no residents reported 0% attention and engagement, and none were engaged for only 25% of the lectures. Four (20%) were engaged for 50%, 13 (65%) for 75%, and 3 (15%) for 100%. This improvement in engagement was statistically significant (*P* = 0.008) (Figure 1).

**Figure 2:**
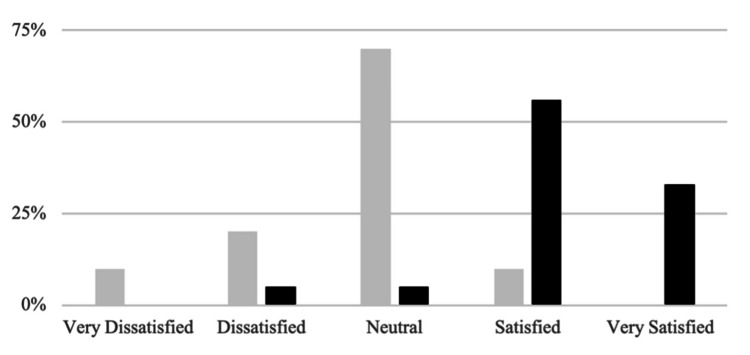
Satisfaction with traditional didactic format compared to split review format. Gray, pre-intervention; black, post-intervention

Regarding the new split format, none were very dissatisfied; 1 (5%) was dissatisfied, 1 (5%) neutral, 11 (56%) satisfied, and 7 (33%) very satisfied. This increase in resident satisfaction was statistically significant (*P* < 0.001) (Figure 2). In the didactic format survey, 19 (95%) respondents reported preferring the new split format over traditional lectures. Additionally, 17 (89%) felt that the new format better prepared them for oral board-style questioning.

The first-time pass rate for the ABS-QE was 11 (100%) pre-change and 9 (90%) post-change (*P* = 0.4762). For the ABS-CE, the first-time pass rate was 6 (55%) pre-change compared to 8 (80%) post-change (*P* = 0.3615).

## Discussion

The split review format combines traditional lectures for ABSITE and ABS-QE preparation with oral board-style questioning for ABS-CE, utilizing a *flipped classroom* model. This approach offers a more comprehensive method for board exam preparation. By limiting each session to 20-25 minutes, it aligns better with adult learner attention spans [6,7]. Our institutional didactic change led to increased resident engagement and satisfaction, with the majority preferring the new format and feeling better prepared for oral board-style questioning. This is consistent with flipped classroom education in other specialties showing increased engagement and satisfaction [5,11-13]. The study showed an improvement in the ABS-CE first-time pass rate; the pre-change group had a 55% pass rate, while post post-change group had an 80% pass rate. However, given the small sample size, we were unable to show statistically significant differences in the first-time pass rates for the ABS-QE or ABS-CE.

A key consideration in designing this format was whether to dedicate the full hour to oral scenarios. However, a split-session approach better aligns with the average adult learner’s attention span. Additionally, much of the content tested on the ABSITE and ABS-QE is not well-suited for oral board-style questioning. The combined format ensures a more comprehensive preparation, effectively equipping residents for both the ABS-QE and ABS-CE.

After implementing this new format for two years, we have identified areas for improvement. The primary concern is the quality of the oral scenarios. While some have been highly effective and engaging, others have been lackluster, largely depending on the individual resident writing the scenario. Since residents have not personally taken the ABS-CE, the focus of their scenarios has not always aligned with the realistic questioning they might encounter or the important aspects that should be highlighted. Going forward, we plan to have faculty surgeons create and lead the scenarios for senior residents. This will offer two benefits: first, faculty surgeons are more familiar with what to expect and can better guide the questioning, and second, senior residents, with their greater knowledge base, will be more capable of handling the scenarios and will benefit most from the practice as they approach the exam. However, not all programs may have the resources or faculty engagement to have faculty surgeons lead questioning regularly. Smaller programs with fewer faculty may struggle to assign a faculty member every week. Programs will need to adapt formats based on their resources, but it seems that senior residents leading junior residents through scenarios would still be more beneficial than traditional lectures alone.

Another potential area for improvement would be the implementation of official case scenarios. Rather than having a resident or even a faculty surgeon design scenarios every week, premade scenarios could serve a large benefit. Being premade, the scenarios would be of higher quality and continually updated and improved over time. It would also reduce the time required every week to generate the material. This could be done through a bank of scenarios generated by the individual programs themselves. Potentially, SCORE could produce an official set of weekly oral scenarios to correlate with their TWIS program. In this way, official scenarios could be provided to programs and utilized in the method that they see best fit.

Limitations

This review has several limitations. First, the study was underpowered to detect significant differences in exam performance. Given our sample size, a 50% difference in first-time pass rates would have been required to achieve statistical significance. To address this, we plan to conduct a follow-up longitudinal study with a larger sample size to better assess the impact on ABS-CE and ABS-QE exam performance. Additionally, residents in the post-change group had been exposed to both educational styles. While this made them well-suited to subjectively evaluate both approaches, it also introduced a confounding factor in the qualitative portion of the study. Furthermore, the presence of COVID-19 during the study period added another confounding variable, particularly in its early stages, as it limited in-person educational sessions and delayed some residents’ ability to take their ABS examinations. Finally, given the predominantly qualitative nature of this study, inherent biases such as selection bias and recall bias must be considered in the study design.

## Conclusions

The split review format has the benefit of integrating both traditional lectures, to prepare for the ABSITE and ABS-QE, and oral board-style questioning to prepare for the ABS-CE, within a *flipped classroom* model. It provides a better format to more completely prepare for both board examinations. It better aligns with the adult learner's attention span and increases engagement and satisfaction. Residents preferred the new format and subjectively felt better prepared for oral board-style questioning. A significant amount of emphasis is placed on ABSITE preparation, and indirectly, on the ABS-QE. With significantly lower ABS-CE pass rates, programs should devote more resources to preparing residents for both exams. Split reviews may be able to more completely prepare residents for their board examinations. This study starts to lay the groundwork for programs to adapt their education. 

## Appendices

Appendix

Pre-intervention Survey

How satisfied are you with our current TWIS review format?”

1-       Very dissatisfied

2-       Dissatisfied

3-       Neutral

4-       Satisfied

5-       Very Satisfied

What percentage of TWIS reviews keep your attention and engagement?”

1-       0% (never pay attention)

2-       25% (occasionally listen but often focus on other tasks)

3-       0% (engaged in about half of the reviews)

4-       75% (usually pay attention but sometimes get distracted)

5-       100% (fully engaged in every review)

Post-intervention Survey

How satisfied are you with our current TWIS review format?”

6-       Very dissatisfied

7-       Dissatisfied

8-       Neutral

9-       Satisfied

10- Very Satisfied

What percentage of TWIS reviews keep your attention and engagement?”

6-       0% (never pay attention)

7-       25% (occasionally listen but often focus on other tasks)

8-       0% (engaged in about half of the reviews)

9-       75% (usually pay attention but sometimes get distracted)

10- 100% (fully engaged in every review)

Which TWIS review format do you prefer?

-              Traditional PowerPoint or new split format

Do you feel these reviews better prepare you for oral board-style questioning

-              Yes or No
